# Spinal Extradural Solitary Fibrous Tumor Arising at the Level of T12 Vertebra: A Rare and Challenging Diagnosis

**DOI:** 10.7759/cureus.90358

**Published:** 2025-08-18

**Authors:** Aashima Prakashe, Ninad Shrikhande, Sagarika S Bhole

**Affiliations:** 1 General Surgery, NKP Salve Institute of Medical Sciences, Nagpur, IND; 2 Neurosurgery, NKP Salve Institute of Medical Sciences, Nagpur, IND

**Keywords:** extradural tumor, mesenchymal tumor, rare spinal tumor, solitary fibrous tumor, spinal neoplasm, spinal soft tissue tumor

## Abstract

Solitary fibrous tumors (SFTs) are uncommon mesenchymal neoplasms that rarely involve the central nervous system (CNS). Their extradural occurrence in the spinal region is exceptionally rare and often mimics more common lesions such as nerve sheath tumors, posing a diagnostic challenge. A case is presented of a young female patient with a gradually progressive swelling in the lower back associated with bilateral lower limb pain. Initial radiological findings suggested a benign neural lesion. Advanced imaging revealed an extradural mass arising from the thoracic spine with features suggestive of a nerve sheath tumor. The mass was surgically excised in its entirety, and subsequent histopathological and immunohistochemical analysis confirmed the diagnosis of a solitary fibrous tumor. No adjuvant therapy was required, and the patient remained disease-free on short-term follow-up. This case underscores the importance of considering SFTs in the differential diagnosis of spinal lesions and highlights the role of surgical excision and histopathological confirmation in management.

## Introduction

Solitary fibrous tumors (SFTs) are rare spindle cell neoplasms of mesenchymal origin, initially described in the pleura but now recognized to occur in various extrapleural sites, including the peritoneum, soft tissues, orbit, and central nervous system (CNS) [1,2]. They are believed to arise from fibroblastic or myofibroblastic cells and are characterized histologically by a "patternless pattern" of cellularity and prominent vascularity [3]. While pleural SFTs account for the majority of cases, extrapleural SFTs have garnered increasing attention due to their diverse anatomical presentations and diagnostic complexity.

Involvement of the spinal axis by SFTs is extremely rare, with few cases reported in the literature. Within the spine, SFTs can occur intramedullary, intradural-extramedullary, or extradural in location, with extradural spinal SFTs representing an uncommon and underrecognized entity [4]. These tumors typically present as slow-growing masses causing compressive symptoms such as back pain, radiculopathy, or myelopathy depending on their location and size.

The radiologic features of spinal SFTs are often nonspecific and can closely resemble more common spinal tumors such as schwannomas or meningiomas, leading to diagnostic confusion [5]. MRI findings may show iso- to hypointensity on T1-weighted images and variable T2 signal intensity, with enhancement postcontrast administration. Because preoperative differentiation from other neoplasms is challenging, definitive diagnosis relies on histopathological examination and immunohistochemical staining, particularly for STAT6 nuclear expression, a highly specific marker for SFTs resulting from NAB2-STAT6 gene fusion [6].

Despite being largely benign, SFTs exhibit a wide spectrum of biological behavior, with potential for local recurrence and rare malignant transformation, emphasizing the need for complete surgical excision and vigilant follow-up [7].

We report a rare case of a spinal extradural SFT located at the T12 vertebral level. The clinical presentation, imaging characteristics, histopathological features, and early postoperative outcome are discussed, highlighting the diagnostic and therapeutic considerations pertinent to this unusual tumor.

## Case presentation

History and clinical examination

**Figure 1:**
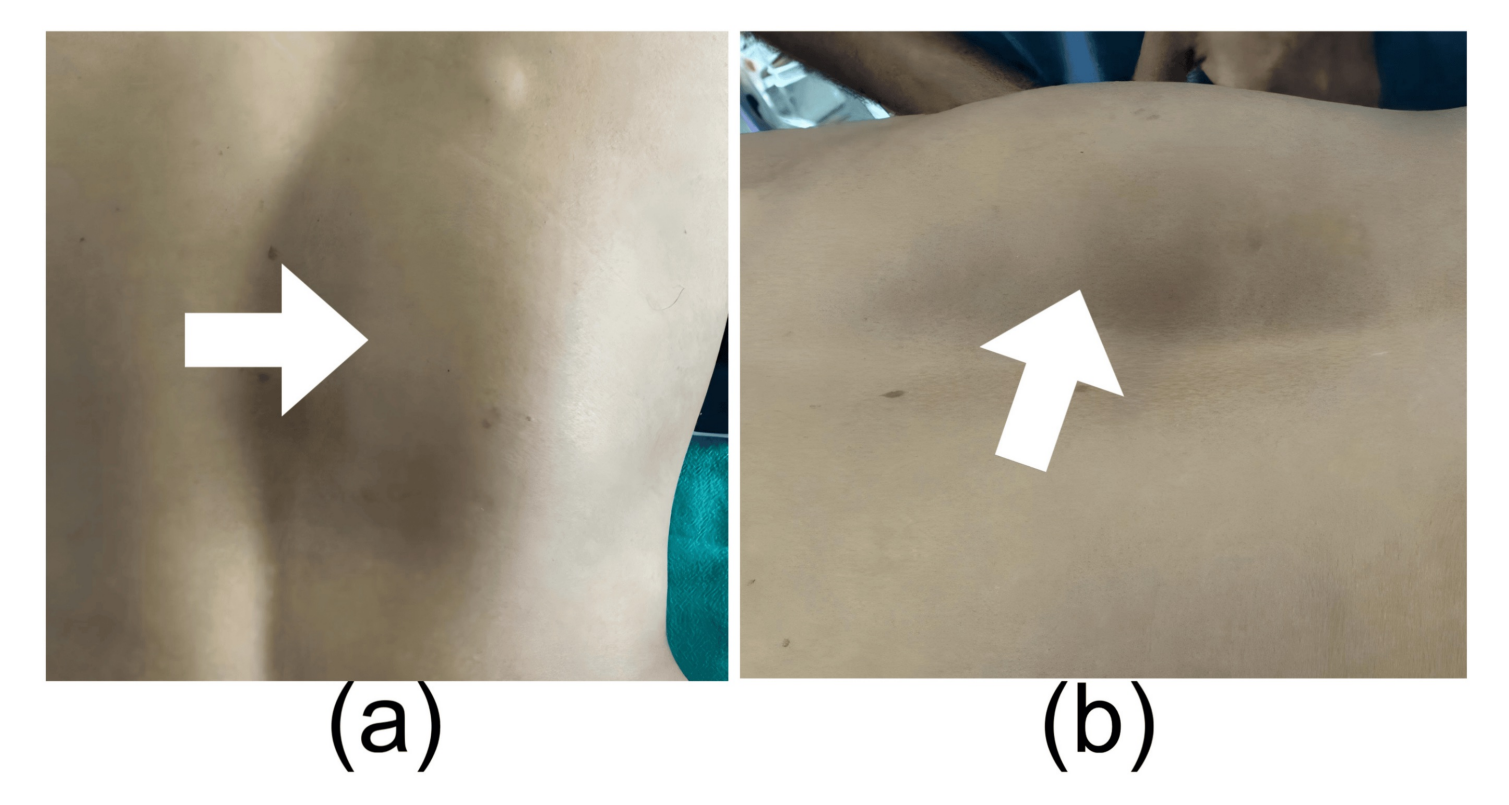
Clinical photograph showing the swelling over right paraspinal region. (a) Posterior view reveals a well-defined, localized swelling over the right paraspinal region. (b) Lateral view demonstrates the extent and projection of the swelling

A 30-year-old woman presented with a 12-month history of a gradually progressive, enlarging swelling over the lower back, which was insidious in onset and associated with bilateral lower limb pain. Clinically, a swelling of 12 x 8 cm was palpated, a firm, nontender swelling, in the right paraspinal region (Figure 1).

An additional secondary swelling measuring 2 × 2 cm was noted beneath the right scapula. It was a firm swelling with a punctum. Based on clinical evaluation, it was diagnosed as a sebaceous cyst; however, priority was given to addressing the primary swelling.

Neurological examination demonstrated increased muscle tone in bilateral lower limbs with a positive Babinski sign bilaterally. She had a normal gait. Upper limb examination was normal. Her past medical history was unremarkable; there was no history of trauma or tuberculosis, and there was no family history of central nervous system disorders.

Investigations

**Figure 2:**
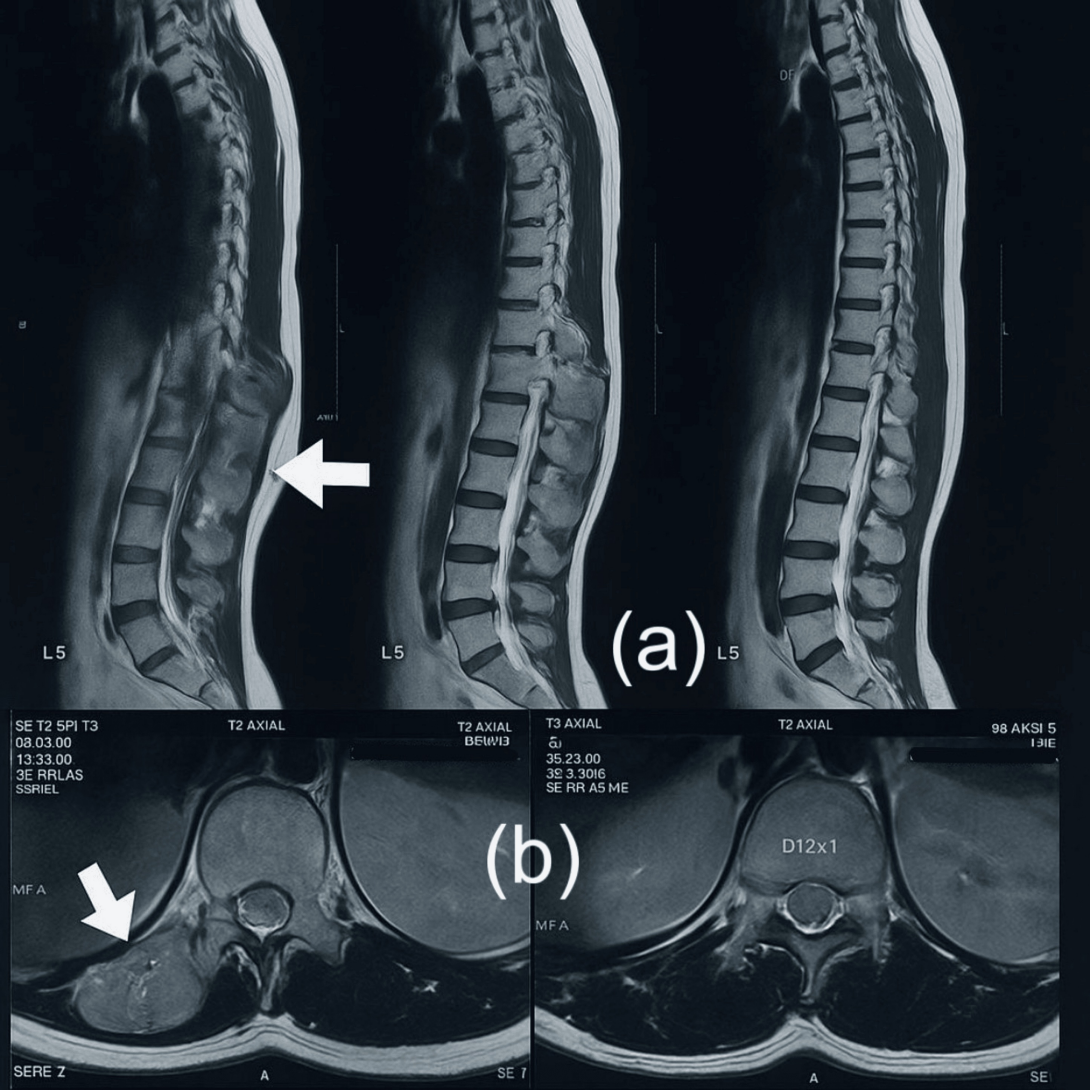
Sagittal and axial T2-weighted MRI images showing a heterogeneously enhancing extradural mass at T12 (white arrows), arising from the right neural foramen. The lesion extends into the right erector spinae muscle and causes bony erosion of the lamina, pedicle, and transverse process-mimicking a nerve sheath tumor (a) Sagittal T2-weighted MRI images of the thoracolumbar spine show a heterogeneously hyperintense extradural mass at the T12 level (white arrow), arising from the right neural foramen. (b) Axial T2-weighted MRI images demonstrate the lesion extending into the right erector spinae muscle (white arrow), with compression of the spinal canal and features resembling a nerve sheath tumor

Routine laboratory investigations were within normal limits. Ultrasonography suggested a benign soft tissue lesion. Contrast-enhanced computed tomography (CT) of the abdomen revealed an ill-defined, heterogeneously enhancing extramedullary, extradural soft tissue mass measuring approximately 3.5 × 5.5 × 10.9 cm (transverse × anteroposterior × craniocaudal). The lesion appeared to originate from the right neural foramen of the T12 vertebra, leading to foraminal widening and extending into the intramuscular plane of the right erector spinae muscle (Figure 2). There was associated bony erosion involving the right lamina, pedicle, and transverse process of the T12 vertebra. A nonenhancing necrotic component was noted at the caudal end of the lesion. No calcifications were identified. The radiological findings were suggestive of a nerve sheath tumor.

Surgical management

**Figure 3:**
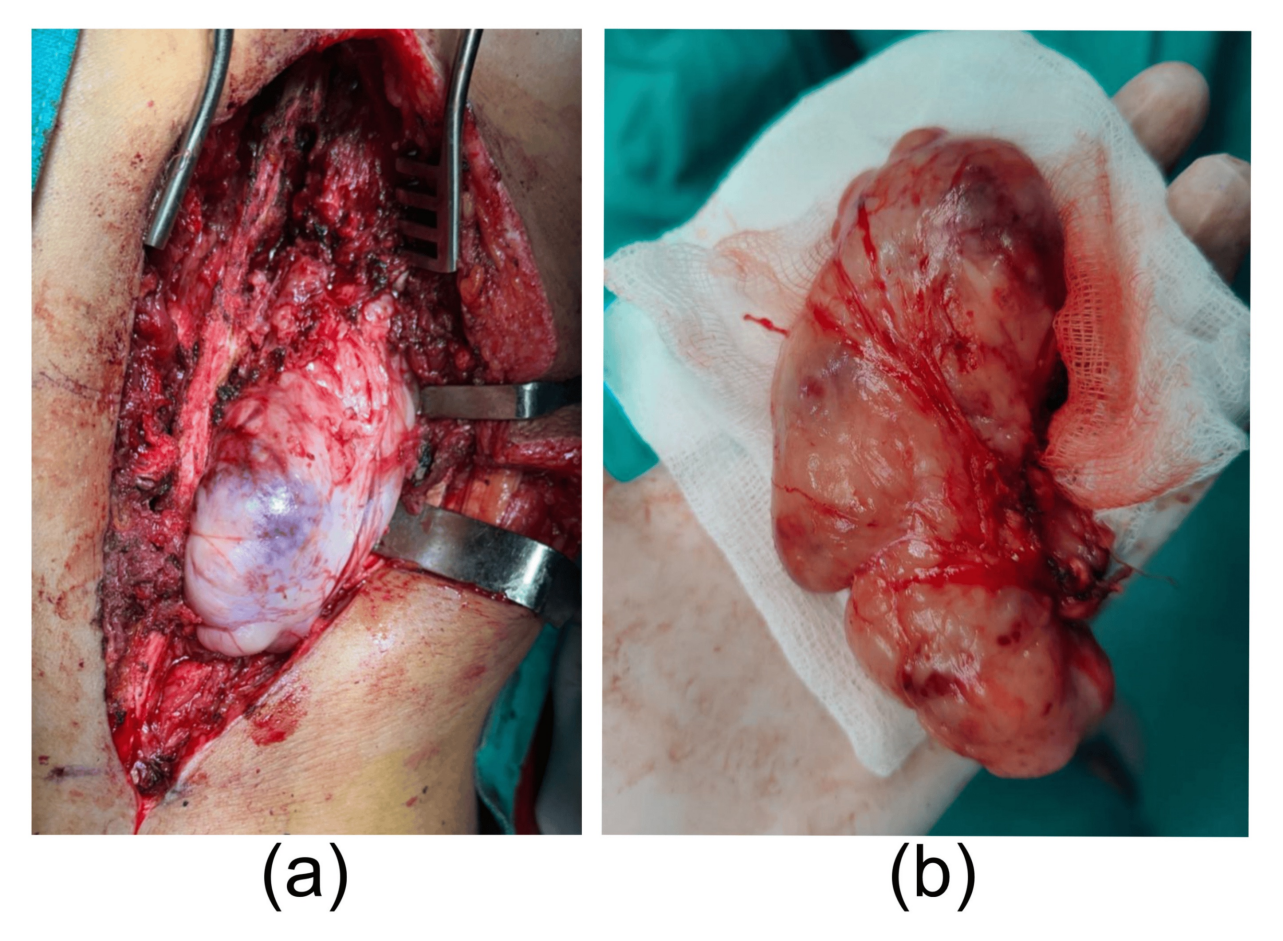
(a) Intraoperative view of a well-encapsulated extradural tumor at T12. (b) Excised lobulated mass of 12 x 8 cm with a glistening surface and focal hemorrhagic areas

A preoperative diagnosis of neurofibroma was made. The patient underwent surgical excision via a posterior midline approach. Intraoperatively, a well-circumscribed, whitish-yellow extradural tumor of approximately 12 x 8 cm was identified. A distinct cleavage plane was present between the tumor and the spinal cord. Gross total resection of the mass was achieved (Figure 3). The secondary swelling of 2 x 2 cm was also excised with its capsule and sent for histopathological evaluation.

Histopathological findings

Microscopic examination revealed a partially encapsulated tumor composed of spindle to ovoid monomorphic cells arranged in a disorganized pattern. The tumor stroma contained hyalinized, dilated, thin-walled, branching blood vessels. The neoplastic cells exhibited round to oval nuclei with scant cytoplasm and minimal pleomorphism. Sparse collagenous stroma and occasional mitoses were observed, with no significant necrosis or hemorrhage. The histological features were consistent with a Grade 1 solitary fibrous tumor. Ki-67 proliferation index was less than 5%. Immunohistochemistry was positive for STAT6, CD34, and BCL2 and negative for S100, desmin, and cytokeratin. The histopathological report of the secondary swelling was consistent with a sebaceous cyst.

Postoperative course and follow-up

The postoperative period was uneventful, and the patient showed complete resolution of lower limb pain without any new neurological deficits. At six months follow-up, the patient remained asymptomatic, and magnetic resonance imaging (MRI) demonstrated no evidence of tumor recurrence.

## Discussion

SFTs, previously categorized under hemangiopericytomas, are rare mesenchymal neoplasms that can exhibit either benign or malignant behavior. Although they primarily originate from the pleura, extrapleural manifestations, including those in the central nervous system (CNS), are exceedingly rare and diagnostically challenging due to nonspecific imaging features that often mimic nerve sheath tumors or meningiomas [8,9].

CNS SFTs carry a significant risk of local recurrence and can impact long-term quality of life. The World Health Organization (WHO) classification provides critical prognostic insights, categorizing these tumors into Grades I to III based on mitotic activity, necrosis, and cellular atypia. WHO Grade I tumors are generally benign and may be managed effectively with gross total resection alone. However, Grades II and III tend to behave more aggressively and are associated with higher recurrence and metastatic potential [10].

Adjuvant therapies, including radiotherapy and chemotherapy, have been explored in high-grade or incompletely resected tumors. While radiotherapy may reduce local recurrence rates, especially in Grade II and III SFTs, its effect on overall survival remains inconclusive. Similarly, the role of chemotherapy is not well-established and is typically reserved for progressive or metastatic disease [11].

In the present case, histopathology was consistent with a WHO Grade I SFT. The patient underwent complete surgical excision, and no adjuvant therapy was administered. At the six-month follow-up, the patient remained asymptomatic with no evidence of recurrence. Despite the benign nature of low-grade SFTs, long-term surveillance is essential, as late recurrences have been reported even in cases with complete resection [12].

## Conclusions

SFTs of the spine, particularly in the extradural space, are rare and often mimic more common spinal neoplasms, making preoperative diagnosis challenging. Histopathology with immunohistochemistry, especially STAT6 and CD34, is essential for confirmation. Complete surgical excision with clear margins remains the mainstay of treatment, offering the best long-term control. Although usually benign, the risk of recurrence or malignant transformation warrants prolonged follow-up. Increased awareness of this entity is crucial for timely diagnosis and optimal outcomes.
